# Trigger factor assisted soluble expression of recombinant spike protein of porcine epidemic diarrhea virus in *Escherichia coli*

**DOI:** 10.1186/s12896-016-0268-7

**Published:** 2016-05-04

**Authors:** Da-Chuan Piao, Do-Woon Shin, In-Seon Kim, Hui-Shan Li, Seo-Ho Oh, Bijay Singh, S. Maharjan, Yoon-Seok Lee, Jin-Duck Bok, Chong-Su Cho, Zhong-Shan Hong, Sang-Kee Kang, Yun-Jaie Choi

**Affiliations:** Department of Agricultural Biotechnology and Research Institute for Agriculture and Life Sciences, Seoul National University, Seoul, 08826 Republic of Korea; Institute of Green-Bio Science & Technology, Seoul National University, Pyeongchang-gun, 25354 Republic of Korea; Department of Animal Science, Tianjin Agricultural University, Tianjin, 300-384 People’s Republic of China

**Keywords:** PEDV, Spike glycoprotein, Subunit vaccine, Inclusion bodies, Chaperone co-expression system, Trigger factor

## Abstract

**Background:**

Porcine epidemic diarrhea virus (PEDV) is a highly contagious enteric pathogen of swine. The spike glycoprotein (S) of PEDV is the major immunogenic determinant that plays a pivotal role in the induction of neutralizing antibodies against PEDV, which therefore is an ideal target for the development of subunit vaccine. In an attempt to develop a subunit vaccine for PEDV, we cloned two different fragments of S protein and expressed as glutathione S-transferase (GST)-tagged fusion proteins, namely rGST-COE and rGST-S1D, in *E.coli*. However, the expression of these recombinant protein antigens using a variety of expression vectors, strains, and induction conditions invariably resulted in inclusion bodies. To achieve the soluble expression of recombinant proteins, several chaperone co-expression systems were tested in this study.

**Results:**

We firstly tested various chaperone co-expression systems and found that co-expression of trigger factor (TF) with recombinant proteins at 15 °C was most useful in soluble production of rGST-COE and rGST-S1D compared to GroEL-ES and DnaK-DnaJ-GrpE/GroEL-ES systems. The soluble rGST-COE and rGST-S1D were purified using glutathione Sepharose 4B with a yield of 7.5 mg/l and 5 mg/l, respectively. Purified proteins were detected by western blot using mouse anti-GST mAb and pig anti-PEDV immune sera. In an indirect ELISA, purified proteins showed immune reactivity with pig anti-PEDV immune sera. Finally, immunization of mice with 10 μg of purified proteins elicited highly potent serum IgG and serum neutralizing antibody titers.

**Conclusions:**

In this study, soluble production of recombinant spike protein of PEDV, rGST-COE and rGST-S1D, were achieved by using TF chaperone co-expression system. Our results suggest that soluble rGST-COE and rGST-S1D produced by co-expressing chaperones may have the potential to be used as subunit vaccine antigens.

**Electronic supplementary material:**

The online version of this article (doi:10.1186/s12896-016-0268-7) contains supplementary material, which is available to authorized users.

## Background

Porcine epidemic diarrhea (PED) is a highly infectious and contagious enteric disease of swine [1]. The disease is characterized by severe diarrhea, vomiting, dehydration and death, with a high mortality rate of more than 90 % in suckling piglets [2]. In the early 1970s, the first case of PED was reported in England [3]. Since then, the disease has spread to Europe and most of the Asian swine raising countries, and severely affected swine industry in Asia [4, 5]. In 2013, PED suddenly occurred in the United States and rapidly spread across the country [6], as well as neighboring countries [7, 8], and subsequently in Asian countries including South Korea [9], Japan and Taiwan [10], and led to significant economic losses in the global swine industry [11]. Thus, it is important to develop an effective vaccine for the prevention of PED.

A coronavirus named porcine epidemic diarrhea virus (PEDV) was identified as the causative agent of PED in the late 1970s [2]. The virus possesses four structural proteins including 150–220 kDa spike (S) glycoprotein, 7 kDa envelop (E) protein, 20–30 kDa membrane (M) protein and 58 kDa neucleocapsid (N) protein [12]. The S protein is a transmembrane glycoprotein localized on the virion surface, which can be divided into the S1 (aa 1–789) domain and S2 (aa 790–1383) domain [13, 14]. The S protein plays a pivotal role in the cellular receptor binding, viral fusion, and most importantly in the induction of neutralizing antibodies [15, 16]. A neutralizing epitope region, COE (aa 499–638), corresponds to the neutralizing epitope of transmissible gastroenteritis virus (TGEV), was identified based on the nucleotide sequence homology analysis [17]. Subsequently, a novel neutralizing epitope region S1D (aa 636–789) on the S1 domain was reported to have the capacity to induce neutralizing antibodies against PEDV [13]. In addition, a B-cell epitope 2C10 (aa 1,368–1,374) on the S2 domain was also reported to induce neutralizing antibodies [18]. Therefore, spike protein is considered as a primary target for the development of subunit vaccine against PEDV.

Up to the present, a number of expression systems have been used to produce S protein of PEDV, such as mammalian cells [16], transgenic plant [19], yeast [20] and *E.coli* [13, 17]. Despite absence of post-translational modifications (such as phosphorylation, acetylation, and glycosylation), expression of recombinant proteins in *E. coli* has many significant benefits over other expression systems in terms of cost, ease-of-use, and scale [21]. However, recombinant protein overexpression in *E.coli* often leads to the misfolding of the protein of interest into biologically inactive aggregates known as inclusion bodies (IBs) [22]. Various expression approaches have been suggested to prevent IBs formation, including utilization of solubility enhancing tag, lowering induction temperature, modulating inducer concentration and changing specialized expression hosts [23]. Moreover, many refolding methods have also been explored for the recovery of soluble proteins from purified IBs [24, 25]. However, these approaches are not always effective, since even the most robust protocols only refold a small fraction of the input protein, and it is difficult to purify the refolded fraction [26]. As an alternative strategy, chaperone co-expression strategies have been proposed to facilitate soluble expression of recombinant proteins with success for many different types of proteins [27–29]. Chaperones are complex molecular machinery that assists the folding of newly synthesized proteins to the native state and provide a quality control system that refolds misfolded and aggregated proteins [30, 31]. In the *E.coli* cytosol, a ribosome-associated folding catalyst known as trigger factor and molecular chaperone teams such as the DanK-DnaJ-GrpE and the GroEL-GroES are profoundly involved in the folding and refolding process [32–35].

In the present study, we report the results of the chaperone-assisted production of soluble rGST-COE and rGST-S1D in *E.coli* as well as the evaluation of immunogenicity and efficacy of purified recombinant proteins as a subunit vaccine antigen.

## Results and discussion

### Chaperone assisted expression of soluble rGST-COE and rGST-S1D

Based on the sequence information of the spike protein of PEDV CV777 strain, we constructed pG-COE and pG-S1D expression plasmids, producing rGST-COE (containing 138 amino acids spanning the region of 499–636 amino acids) [17] and rGST-S1D (containing 153 amino acids spanning the region of 637–789 amino acids) [13], respectively. However, unlike the previous reports [13, 17], our initial attempts to produce rGST-COE and rGST-COE using a pGEX 6p-1 vector system with various induction conditions resulted in IBs (data not shown). Since, both of the COE and S1D regions contain 4 cysteine residues in the protein sequence, we also performed expression of these clones in *E.coli* Rosetta-gami™2 (DE3) (Novagen, Darmstadt, Germany) strain known to enhance disulfide bond formation in the cytoplasm, although the yield of soluble proteins was negligible (data not shown).

**Fig. 1:**
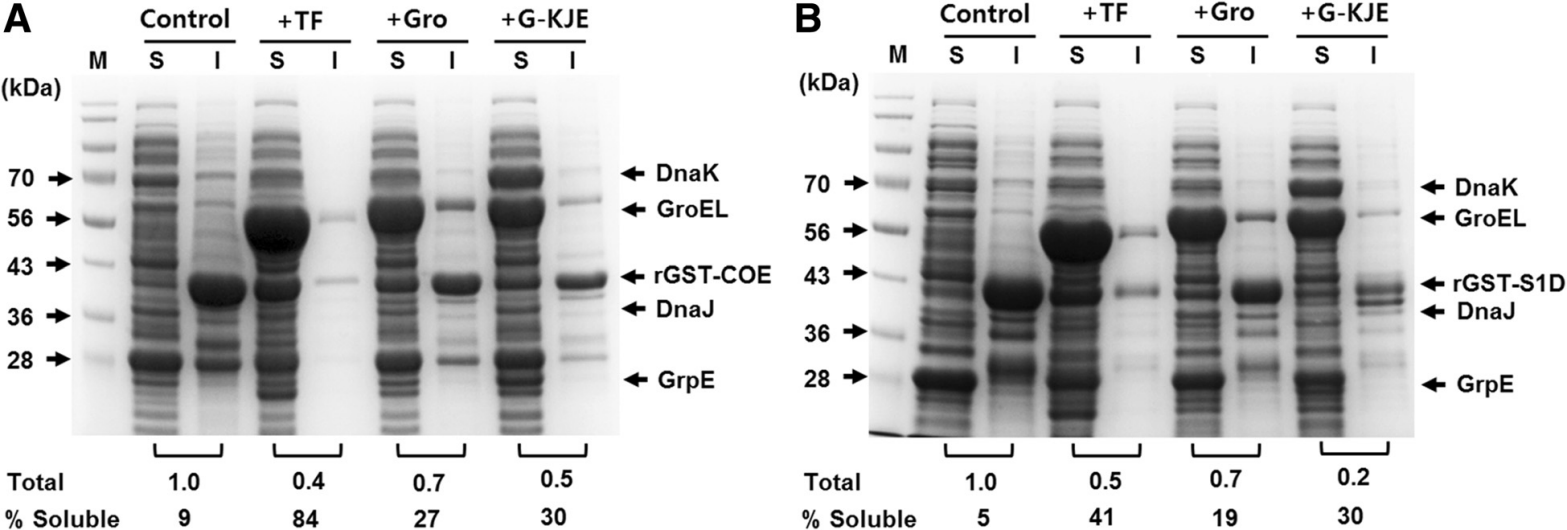
Chaperone assisted expression of soluble rGST-COE (**a**) and rGST-S1D (**b**). Chaperone proteins were induced at 37 °C by adding L-arabinose (0.5 mg/ml) or tetracycline (10 ng/ml) prior to the expression of recombinant proteins. Target proteins were induced by adding 0.1 mM IPTG at 15 °C for 24 h. Soluble (S) and insoluble (I) fractions were analyzed by SDS-PAGE. The protein bands for rGST-COE, rGST-S1D and chaperone proteins are indicated on the right side of the gel. Lanes: control, no chaperone; +TF, co-expression with trigger factor; +Gro, co-expression with GroEL-GroES complex; +G-KJE: co-expression with DnaKJE/GroEL-GroES complex

Previously, chaperone co-expression system has been reported to improve the solubility of various aggregation-prone recombinant proteins, such as human interferon-gamma, mouse endostatin, human ORP150 and human lysozyme [27, 36]. Therefore, we decided to test the effect of chaperone systems, such as trigger factor (TF), GroEL-ES, DnaKJE/GroEL-ES and GroEL-ES/TF, on the production of soluble rGST-COE and rGST-S1D. The pG-COE and pG-S1D expression plasmids were transformed in to the *E.coli* BL21, BL21/pTF16, BL21/pGro7, BL21/pG-KJE8 and BL21/pG-Tf2 chaperone competent cells. As shown in Fig. 1, we found that co-expression of TF with recombinant proteins at 15 °C was most useful in soluble production of rGST-COE and rGST-S1D compared to other chaperone combinations (Fig. 1). Majority of the recombinant proteins were presented in insoluble fraction when expressed alone, while co-expression of recombinant proteins with TF resulted in up to 84 % soluble fraction for the rGST-COE, and 41 % for the rGST-S1D compared to 19 ~ 30 % of other chaperone systems. Total amount of recombinant proteins expressed were decreased to 40 ~ 50 % value of no chaperone induction. Interestingly, TF system is a cold shock chaperone, while the GroEL-ES and the DnaK-DnaJ-GrpE systems belong to the heat shock chaperones and the ratios of chaperone combinations are critical in the folding or refolding process [30, 37]. In this study as we used commercial system, the ratios of chaperones or other conditions were not optimized for the PEDV antigen expression. Nonetheless, TF system is simple and most effective among the tested conditions for the soluble expression of PEDV antigens and do not require co-chaperones or ATP as compared to the GroEL-ES and the DnaK-DnaJ-GrpE systems.

Furthermore, TF, a ribosome associated chaperone, is the first chaperone that interacts with nascent polypeptide chains and assists co-translational protein folding [38]. Thus, TF may be essential for the correct folding of rGST-COE and rGST-S1D during initial *de novo* folding steps [27, 39]. It was reported that formation of GroEL/TF complex can further enhance binding affinity of GroEL to unfolded protein and facilitate protein folding or denaturation. However, co-expression of GroES-GroEL/TF system (*E.coli* BL21/pG-Tf2) was not successful in our case due to the growth inhibition observed upon chaperone induction (data not shown).

**Fig. 2:**
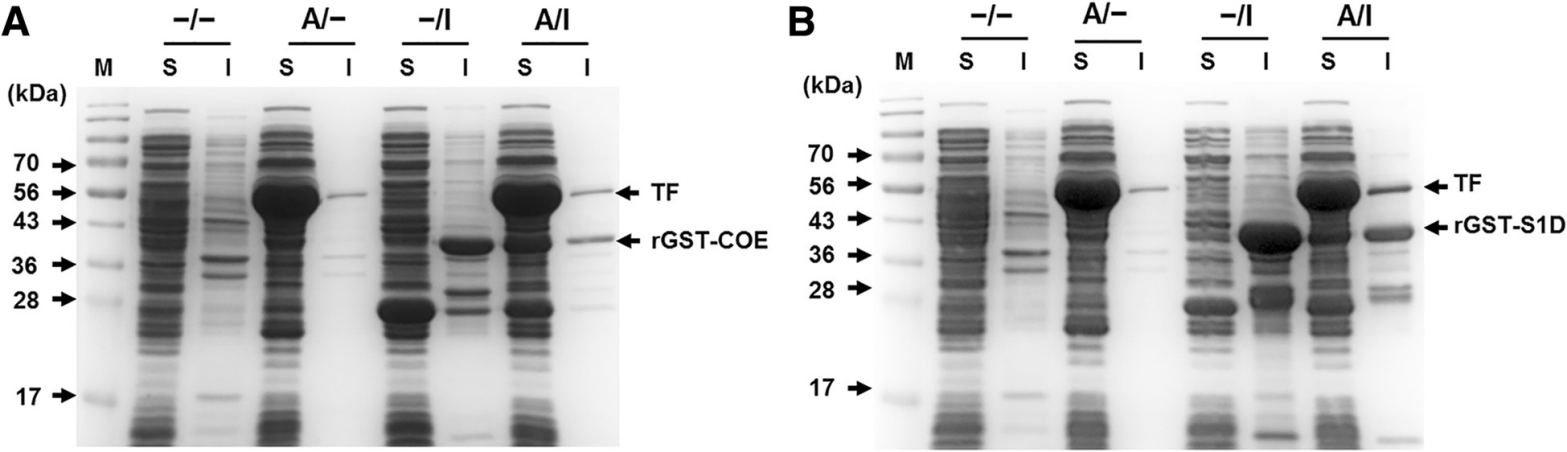
Effect of trigger factor (TF) on the solubility of rGST-COE (**a**) and rGST-S1D (**b**). TF was induced prior to the expression of recombinant proteins by adding L-arabinose (0.5 mg/ml) at 37 °C. Target proteins were induced by adding 0.1 mM IPTG at 15 °C for 24 h. Soluble (S) and insoluble (I) fractions were analyzed by SDS-PAGE. The protein bands for rGST-COE, rGST-S1D and TF are indicated. Lanes: −/−, no induction; A/-, induction with L-arabinose; −/I, induction with IPTG; A/I, induction with L-arabinose and IPTG

As shown in Fig. 2, SDS–PAGE revealed the expression of TF (56 kDa) induced by L-arabinose and the expression of rGST-COE (Fig. 2a) and rGST-S1D (Fig. 2b) induced by IPTG. Recombinant proteins or chaperone TF was not produced without IPTG or L-arabinose induction, respectively. Insoluble recombinant proteins were produced with IPTG in the absence of L-arabinose. Soluble recombinant proteins were produced only in the presence of TF. As a result, it was confirmed that co-expression of TF highly improved the solubility of rGST-COE and rGST-S1D.

### Optimization of expression conditions for soluble rGST-COE and rGST-S1D production

To maximize the soluble expression of rGST-COE and rGST-S1D, expression conditions for *E.coli* BL21/pTf16/pG-COE and *E.coli* BL21/pTf16/pG-S1D were optimized by regulating induction temperature, IPTG concentration, OD at induction and harvest time. The rate of expression and culture temperature can affect the proper folding and IBs formation of most recombinant proteins [40–42]. In order to determine the optimal induction temperature, rGST-COE was induced at different temperatures (15, 21, 28 and 37 °C), while TF was induced with L-arabinose from the culture start. SDS-PAGE results showed that optimal temperature for soluble rGST-COE expression was 15 °C, as the solubility of rGST-COE was increased progressively as temperature decreased from 37 °C to 15 °C. (Additional file 1: Figure S1). Interestingly, the expression level of TF was also increased as the temperature decreased. Indeed, it has been reported that TF, unlike other chaperones in *E.coli*, is a cold-shock chaperone which was induced at low temperature [37]. Our data suggest that the improved solubility of rGST-COE at low temperature may be attributed to the elevated expression level of TF, but not duo to the reduction of expression rate at low temperature.

**Fig. 3:**
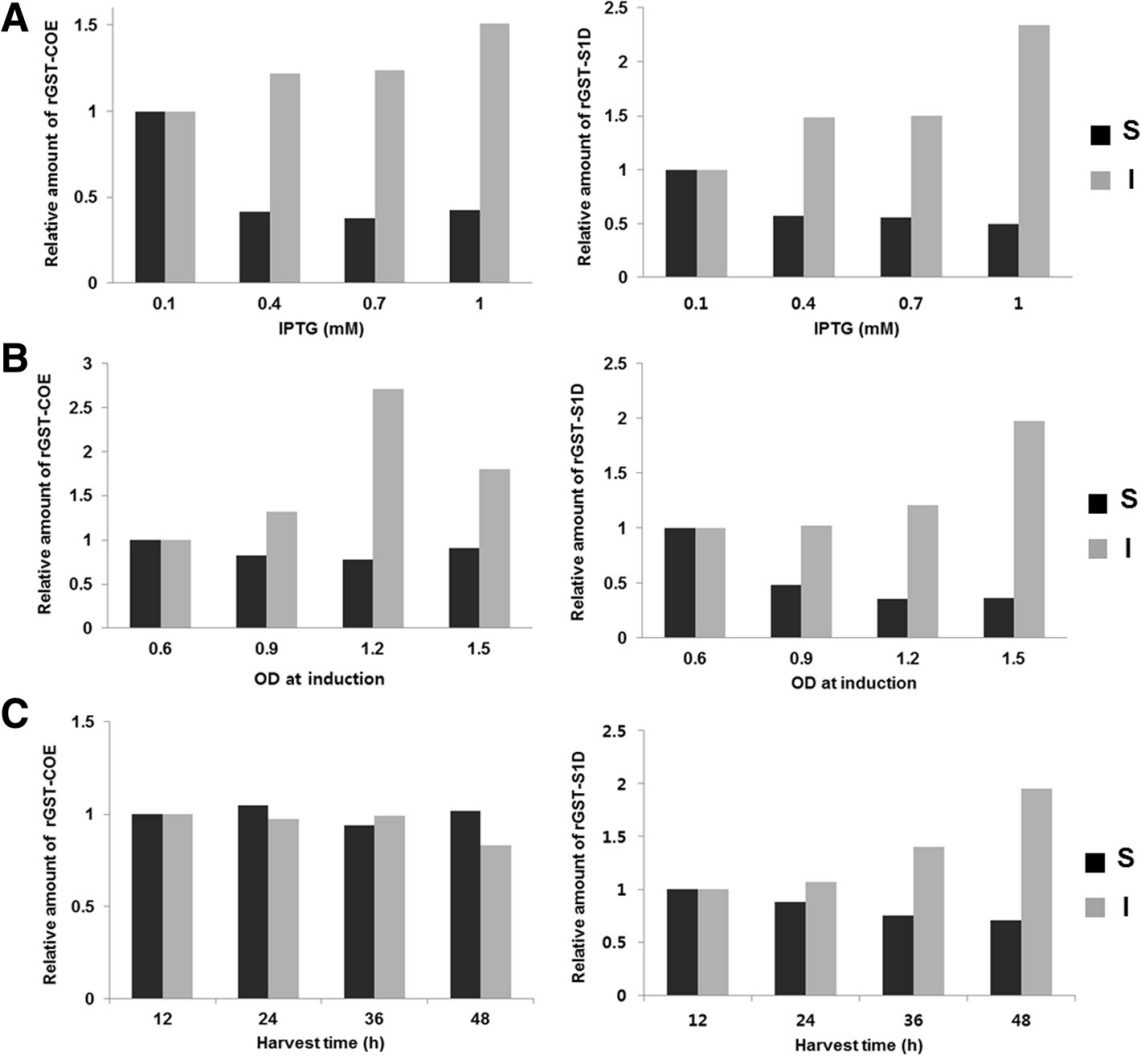
Optimization of IPTG concentration (**a**), OD at induction (**b**) and harvest time (**c**) for soluble rGST-COE and rGST-S1D expression. The amount of soluble (S) and insoluble (I) fractions were compared relatively with each starting condition (IPTG = 0.1 mM, OD = 0.6, Time = 12 h), respectively, according to the band intensities of SDS-PAGE. The experiment was conducted twice independently, and the data from a representative experiment are shown

After determining the optimal expression temperature, the effect of IPTG concentration (0.1, 0.4, 0.7 and 1.0 mM), OD at induction (at OD600 0.6, 0.9, 1.2 and 1.5) and harvest time (12, 24, 36 and 48 h after induction) were examined on the efficiency of soluble expression. Optimal conditions for soluble expression were 0.1 mM IPTG concentration, induction start at OD600 0.6, and induction time of 24 h or 12 h for rGST-COE and rGST-S1D at 15 °C (Fig. 3).

### Purification and western blot confirmation of rGST-COE and rGST-S1D

**Fig. 4:**
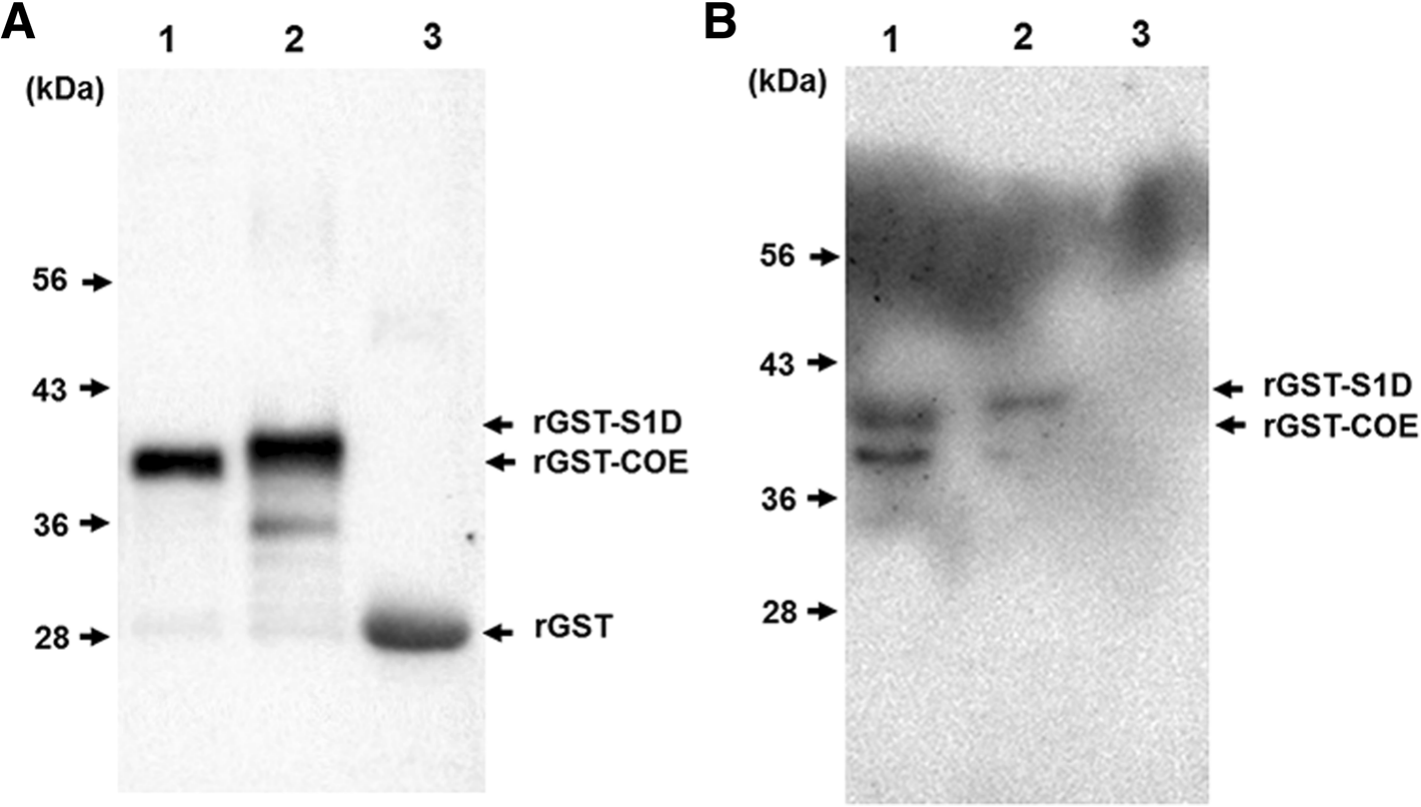
Detection of purified rGST-COE and rGST-S1D proteins by western blot analysis using mouse anti-GST monoclonal antibody (**a**) and pig anti-PEDV immune sera (**b**). Lanes: 1, rGST-COE; 2, rGST-S1D; 3, rGST (Control)

The soluble rGST-COE and rGST-S1D fractions were purified by affinity column chromatography using glutathione Sepharose 4B. The soluble fraction obtained after cell lysis and centrifugation was loaded on the column pre-equilibrated with buffer A (140 mM NaCl, 2.7 mM KCl, 10 mM Na2HPO4 and 1.8 mM KH2PO4 at pH 7.3). The column was washed successively with buffer A. The bound proteins were eluted using buffer B (50 mM Tris–HCl and 10 mM glutathione at pH 8.0) and fractions containing purified proteins were collected and the purity of the protein was analyzed by SDS-PAGE (Additional file 1: Figure S2). When determined by Bradford assay, the yield of purified soluble rGST-COE and rGST-S1D in flask culture were 7.5 mg/l and 5 mg/l, respectively. The purity of the pooled fractions was >90 % and we used them without further purification. 1 μg of purified proteins were separated in the SDS-PAGE (Additional file 1: Figure S3), and target proteins were detected using mouse anti-GST monoclonal antibody and pig anti-sera against commercial PEDV live attenuated vaccine in a western blot analysis (Fig. 4). The rGST protein was included as a control for the western blot analysis. No clear bands were detected with pre-immune sera used as a control in western blot (data not shown). Pig anti-PEDV immune sera showed weak signal, high background and some non-specific reactivity in western blot (Fig. 4b) compared to the ELISA result (Fig. 6). It seems that those two results are not matching to some extent. But, it may be possible to get those results, because the pig anti-PEDV sera was raised against live attenuated virus, which contained polyclonal antibodies recognizing both conformational and linear epitopes on the native PEDV spike protein. Therefore, the pig anti-PEDV sera could detect both conformational and linear epitopes on rGST-COE and rGST-S1D in an ELISA assay, while pig anti-PEDV sera could react only with linear epitopes on denatured rGST-COE and rGST-S1D in a western blot. In this context, it is possible that the reaction in ELISA was much stronger than in western blot.

### Neutralization antibody response of rGST-COE and rGST-S1D

**Fig. 5:**
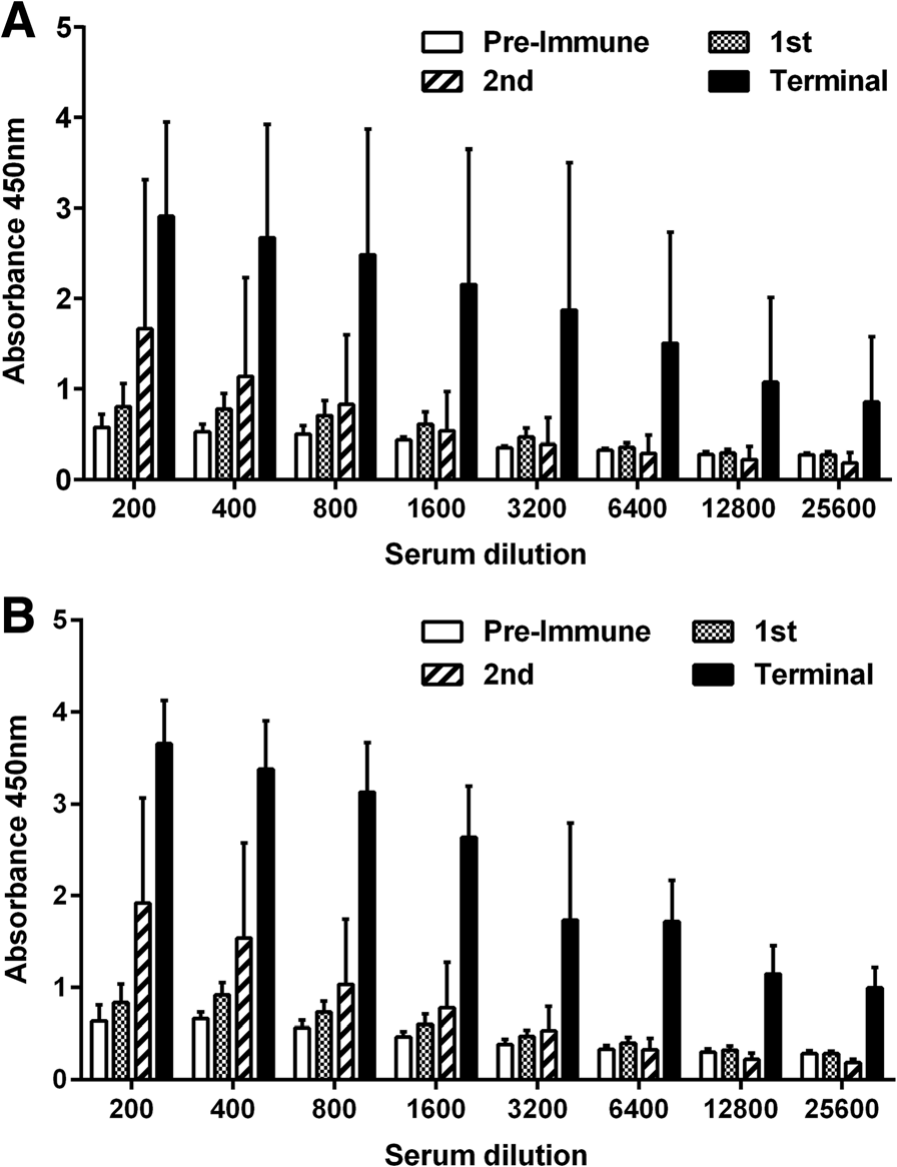
Detection of antigen specific serum IgG levels from mice immunized with rGST-COE (**a**) and rGST-S1D (**b**) by ELISA. Mice (*n* = 5) were immunized subcutaneously with 10 μg of purified proteins three times at 2-weeks intervals. Pre-immune sera and sera from two weeks following each injection were serially diluted and assayed by ELISA. Error bars indicate the standard deviations of the means

**Fig. 6:**
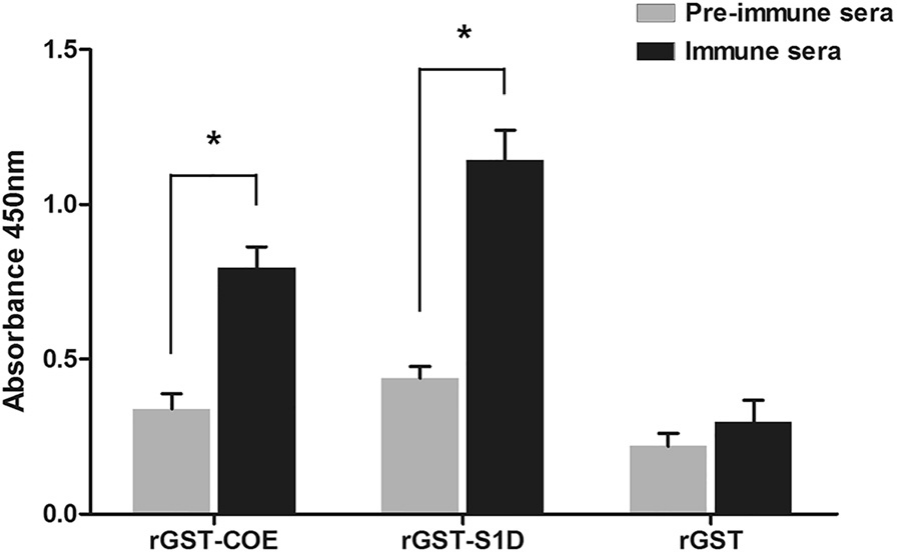
Reactivity of purified rGST-COE and rGST-S1D with pig anti-PEDV immune sera in an indirect ELISA. The rGST was included as a negative control. Error bars indicate the standard deviations of the means. The asterisk indicates the *p* values that are less than 0.05

To determine the immunogenicity of rGST-COE and rGST-S1D, mice were immunized subcutaneously with 10 μg of purified proteins for 3 times at 2-weeks intervals. Mouse anti-sera were collected before and at 14, 28, 42 days after immunization. The 96 well plates were coated with purified rGST-COE or rGST-S1D and reacted with corresponding mouse anti-sera diluted at 1:200 ~ 1:25600 for the detection of antigen specific serum IgG levels. Both rGST-COE and rGST-S1D strongly elicited antigen specific serum IgG production after third injection of antigens (Fig. 5). In the indirect ELISA, rGST-COE and rGST-S1D also showed significant reactions with pig anti-PEDV immune sera (Fig. 6).

**Fig. 7:**
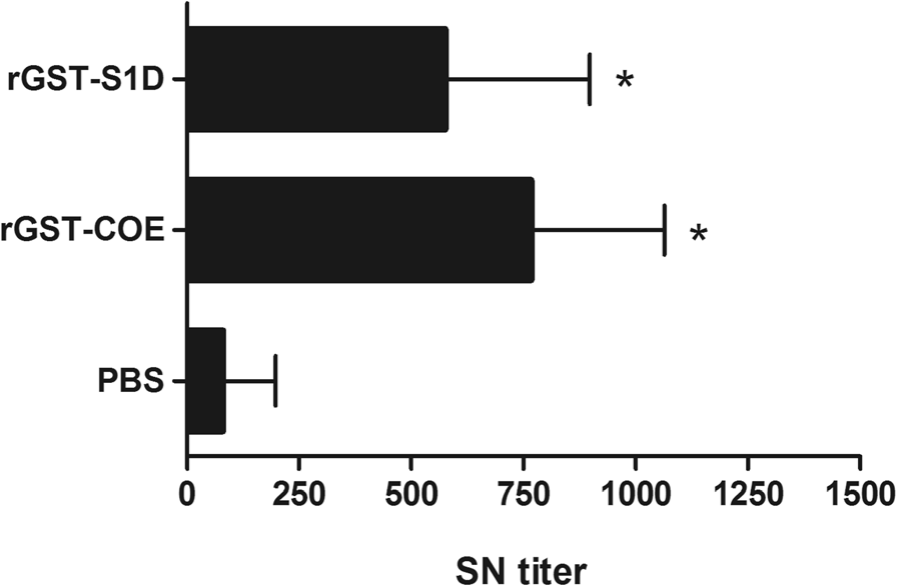
Serum neutralizing (SN) antibody titers from mice immunized with PBS, rGST-COE and rGST-S1D. Terminal serum samples were serially diluted and subjected to the serum neutralization assay. Error bars indicate the standard deviations of the means. The asterisk indicates the p values that were less than 0.05

To test whether immunization of mice with rGST-COE and rGST-S1D could elicit specific neutralizing antibodies against PEDV, terminal serum samples collected from immunized mice were subjected to the serum neutralization assay. As shown in Fig. 7, immunization of mice with rGST-COE and rGST-S1D showed significantly higher SN titer than control mice received PBS.

Even though Sun et al. [13] argued only S1D being effective for the induction of neutralizing antibodies, we confirmed through this study the previous report which showed COE and S1D fragments of PEDV spike protein working as neutralizing epitopes [17]. However, unlike the reports of soluble expression of theses antigens [13, 17], the expression of these recombinant antigens using a variety of expression vectors, strains, and induction conditions invariably resulted in inclusion bodies without proper chaperone co-expression such as TF. Even though we exactly matched the reports in terms of vector, amino acid sequence including GST fusion, *E.coli* strain, and optimization of induction conditions, we could not reproduce the soluble production without the chaperone support.

## Conclusions

In this study, two recombinant fragments of PEDV spike protein, COE and S1D, were produced in soluble forms using a chaperone, TF co-expression system in *E.coli*. Without TF, these antigens invariably resulted in inclusion bodies in variety of expression vectors, host strains, and induction conditions tested. Co-expression of TF with recombinant proteins at 15 °C was most useful in soluble production of rGST-COE and rGST-S1D compared to GroEL-ES and DnaK-DnaJ-GrpE/GroEL-ES systems. The soluble rGST-COE and rGST-S1D were purified and detected by western blot using mouse anti-GST mAb and pig anti-PEDV immune sera, and showed dominant immune reactivity with pig anti-PEDV immune sera in an indirect ELISA assay. Finally, immunization of mice with 10 μg of purified proteins elicited highly potent serum IgG and serum neutralizing antibody titers. Our results suggest that soluble rGST-COE and rGST-S1D may have potential to be used as efficient subunit vaccine antigens for the PEDV prevention. Further studies will be followed to evaluate the efficacy of those recombinant antigens in target animals for the subunit vaccine development.

## Materials and methods

### Strains

The expression plasmids, pG-COE and pG-S1D were constructed by ligating pGEX-6p-1 (GE Healthcare, Uppsala, Sweden) with COE (aa 499–636) and S1D (aa 637–789) regions, respectively, in BamH1 and Xho1 sites. The COE and S1D fragments based on the nucleotide sequence of spike protein of PEDV CV777 strain (GenBank Acc. No. AF353511) were synthesized after codon-optimization by Genscript (Piscataway, NJ, USA). These expression plasmids produced recombinant spike proteins, rGST-COE and rGST-S1D, as glutathione S-transferase (GST)-tagged fusion proteins under the *tac* promoter (*Ptac*). Each plasmid was transformed into *E.coli* BL21, BL21/pTf16, BL21/pGro7, BL21/pG-KJE8 and BL21/pG-Tf2 competent cells (TaKaRa, Japan). *E.coli* BL21/pTf16 strain produced the trigger factor (TF) under the L-arabinose inducible promoter. On the other hand, *E.coli* BL21/pGro7 and BL21/pG-KJE8 strains produced GroEL-GroES and DnaK-DnaJ-GrpE/GroEL-GroES chaperone complex, respectively. *E.coli* BL21/pG-Tf2 strain produced GroEL-GroES/TF complex.

### Recombinant protein expression

A seed culture was prepared by inoculating with a single colony of recombinant *E.coli* strains and grown overnight in 5 ml of LB broth containing 100 μg/ml of ampicillin in a 50 ml sterile plastic tubes. For the expression of recombinant proteins, 1 % seed culture was routinely inoculated to 25 ml of LB broth containing 0.5 mg/ml of L-arabinose, 100 μg/ml of ampicillin and 20 μg/ml of chloramphenicol in 100 ml baffled flasks, followed by adding L-arabinose (0.5 mg/ml) and/or tetracycline (10 ng/ml) for the induction of chaperone proteins. Cells were cultured with shaking at 230 rpm and 37 °C. The growth was monitored by measuring the absorbance of optical density (OD) at 600 nm with a spectrophotometer. When the OD600 of the culture reaches set point, cultures were cooled to 4 °C for 30 min and induced by 0.1 mM IPTG at 15 °C for 24 h.

To check the chaperone effect of trigger factor, *E.coli* BL21/pTf16/pG-COE and *E.coli* BL21/pTf16/pG-S1D were shake-cultured in LB broth at 37 °C until OD600 reached 0.6, cooled to 4 °C for 30 min and induced at 15 °C for 24 h with or without IPTG (0.1 mM) and with or without L-arabinose (0.5 mg/ml) to detect the protein expression.

Cells were harvested and disrupted on ice by sonication (VCX 750, SONICS, Newtown, CT, USA) using a program (45 cycles of 2 sec On/5 s Off, amp 20 %) and centrifuged at 20,000 g for 15 min at 4 °C. The pellets and supernatants were separately stored at −20 °C until use. Protein concentration was measured by Bradford assay (BioRad, CA, USA) using bovine serum albumin (BSA) as a standard. Protein expression was evaluated using band intensities of recombinant protein on sodium dodecyl sulfate polyacrylamide gel electrophoresis (SDS-PAGE) from independent and/or parallel induction samples.

### Optimization of soluble recombinant protein expression

To find the optimal conditions for the soluble expression of recombinant proteins, the effect of various induction conditions for *E.coli* BL21/pTf16/pG-COE and *E.coli* BL21/pTf16/pG-S1D strains were optimized by regulating induction temperature (15 °C for 24 h, 21 °C for 16 h, 28 °C for 8 h and 37 °C for 4 h with 0.1 mM IPTG when OD600 reaches about 0.6), IPTG concentration (0.1, 0.4, 0.7 and 1.0 mM at 15 °C for 24 h shake culture starting at OD600 of 0.6), OD at induction (OD600 = 0.6, 0.9, 1.2 and 1.5 at 15 °C for 24 h shake culture with 0.1 mM IPTG) and harvest time (12, 24, 36 and 48 h after induction at 15 °C with 0.1 mM IPTG).

### Protein purification and analysis

Soluble recombinant proteins were purified from the crude cell extracts by glutathione-affinity chromatography using a glutathione Sepharose 4B (GE Healthcare, Sweden). The column was equilibrated with 5 column volume of binding buffer (140 mM NaCl, 2.7 mM KCl, 10 mM Na2HPO4 and 1.8 mM KH2PO4, pH 7.3) and crude soluble proteins were applied. The column was washed with 5 column volume of binding buffer and bound proteins were eluted with elution buffer (50 mM Tris–HCl and 10 mM glutathione, pH 8.0).

Proteins were analyzed by SDS-PAGE on pre-maid 12 % polyacrylamide mini-gels (KomaBiotech, Korea) run in a Mini-PROTEAN electrophoresis system (Bio-Rad, CA, USA). Gels were stained with Coomassie Blue R250 and images were analyzed by image analysis software of a ChemiDoc™ MP System (BioRad, CA, USA). For the western blot assay, proteins after SDS-PAGE run were transferred to a nitrocellulose membrane (Whatman, USA) in a Mini Trans-Blot system (BioRad, USA). Then the membrane was blocked with Tris-buffered saline/0.1 % Tween 20 (TBST) containing 5 % skim milk (BD/Difco, NJ, USA) for 1 h and probed with mouse anti-GST monoclonal antibody (Genscript, USA) and pig anti-sera against PEDV in TBST at 1:1000 and 1:500 dilutions, respectively, for 1 h both at room temperature. HRP-conjugated goat anti-mouse IgG (MerckMillipore, Germany) and goat anti-porcine IgG (KPL, USA) diluted at 1:5000 were used as secondary antibody. Detection was carried out using an ECL detection kit (GE Healthcare, Sweden).

### Indirect enzyme-linked immunosorbent assay (ELISA)

The reactivity of purified proteins with pig anti-sera against PEDV was tested by an indirect ELISA. The 96 well Immuno-plate (SPL, South Korea) was coated with 5 μg/ml of purified proteins in carbonate buffer (pH 9.6) at 4 °C overnight. Plates were blocked with 1 % bovine serum albumin (BSA) in PBS for 2 h at room temperature. Pre-immune and immune sera were obtained from pigs before and after immunization of commercial PEDV vaccine (Green Cross, South Korea). Sera diluted at 1:1000 were added to the corresponding wells and incubated for 1 h at room temperature. HRP conjugated goat anti-porcine IgG (KPL, USA) at 1:2500 dilution was added and incubated for 1 h at room temperature. Plates were incubated with the TMB substrate solution (Sigma, USA) for 15 min in the dark and reaction was stopped by adding sulfuric acid. Absorbance at 450 nm was measured in an Infinite 200 PRO microplate reader (Tekan, Switzerland).

### Mouse immunization and detection of antibody production

Six-week-old female BALB/c mice were purchased from Samtako (Osan, Korea). The experiments were performed in accordance with the guidelines for the care and use of laboratory animals under the approval of animal ethics committee at Seoul National University (SNU-130415–1). The mice, maintained under standard pathogen-free conditions, were provided with free access to food and water during the experiments. Mice (*n* = 5) were immunized subcutaneously with 10 μg of purified proteins three times at 2-week intervals. Complete Freund’s adjuvant (CFA) and incomplete Freund’s adjuvant (IFA) were mixed with antigens for the priming and boosting of animals. Sera were collected from the mice at 0, 14, 28 and 42 days after first immunization for the antibody detection and serum neutralization assay.

The induction of antigen specific serum IgG level was measured by ELISA. Plates were coated and blocked using the same method described in the indirect ELISA section. After blocking, 2 fold serial diluted mouse sera were added to the wells and incubated for 1 h at room temperature. HRP conjugated goat anti-mouse IgG secondary antibody diluted at 1:5000 was added and incubated for 1 h at room temperature. Other assay procedures are same as described in the indirect ELISA section.

### Serum neutralization (SN) assay

The presence of PEDV specific neutralizing antibodies in the serum collected from immunized mice was determined by a serum neutralizing assay. Terminal serum samples were inactivated at 56 °C for 30 min, followed by 2 fold serial dilutions in a serum-free α-MEM containing 1 % antibiotic-antimycotic solution (Invitrogen, USA). PEDV SM98 isolate of 200 TCID_50_/0.1 ml was mixed with an equal volume of diluted sera. After incubation for 1 h at 37 °C, 0.1 ml of each virus-serum mixture was inoculated onto Vero cell monolayers in the 96-well tissue culture plates. After adsorption for 1 h at 37 °C, virus-serum mixture was removed and plates were washed 3 times with PBS. Serum-free α-MEM medium containing trypsin (2.5 μg/ml) was then added into each well and incubated for 3 ~ 6 days at 37 °C. The SN titers were expressed as the highest serum dilution resulting in the inhibition of cytopathic effect.

### Statistical analysis

The Student’s *t* test was used for all statistical analyses, and *p*-values of less than 0.05 were considered statistically significant.

### Ethics approval

The animal experiments were performed in accordance with the guidelines for the care and use of laboratory animals under the approval of animal ethics committee at Seoul National University (No.: SNU-130415–1).

### Consent for publication

Not applicable.

### Availability of data and material

Not applicable.

## Additional file

Additional file 1: Figure S1. SDS-PAGE analysis of rGST-COE expression at various induction temperatures. S, soluble fraction; I, insoluble fraction. The solid arrows indicate rGST-COE. **Figure S2.** SDS-PAGE analysis of rGST-COE (A) and rGST-S1D (B) purified using glutathione Sepharose 4B. Dotted arrow indicates rGST-COE and rGST-S1D. M, size markers in kDa; Lanes: 1, soluble crude proteins; 2, unbound fractions; 3–4, washing fractions; 5–9, elution fractions. **Figure S3.** SDS-PAGE analysis of purified rGST-COE, rGST-S1D and rGST. 1 μg of purified rGST-COE, rGST-S1D and rGST were loaded on the SDS-PAGE. Lanes: 1, rGST-COE; 2, rGST-S1D; 3, rGST. (DOCX 456 kb)

## Abbreviations

GST: glutathione S-transferase
IPTG: isopropyl β-D-1-thiogalactopyranoside
OD: optical density.
PEDV: porcine epidemic diarrhea virus
TF: trigger factor
