# Supplementary material for: Trigger factor assisted soluble expression of recombinant spike protein of porcine epidemic diarrhea virus in Escherichia coli
Source: BMC Biotechnol. 2016 May 4;16:39. doi: 10.1186/s12896-016-0268-7 (PMC4855837; doi:10.1186/s12896-016-0268-7)
Supplement: Additional file 1: Figure S1. — SDS-PAGE analysis of rGST-COE expression at various induction temperatures. S, soluble fraction; I, insoluble fraction. The solid arrows indicate rGST-COE. Figure S2. SDS-PAGE analysis of rGST-COE (A) and rGST-S1D (B) purified using glutathione Sepharose 4B. Dotted arrow indicates rGST-COE and rGST-S1D. M, size markers in kDa; Lanes: 1, soluble crude proteins; 2, unbound fractions; 3–4, washing fractions; 5–9, elution fractions. Figure S3. SDS-PAGE analysis of purified rGST-COE, rGST-S1D and rGST. 1 μg of purified rGST-COE, rGST-S1D and rGST were loaded on the SDS-PAGE. Lanes: 1, rGST-COE; 2, rGST-S1D; 3, rGST. (DOCX 456 kb) [file 12896_2016_268_MOESM1_ESM.docx]

**Additional file:**


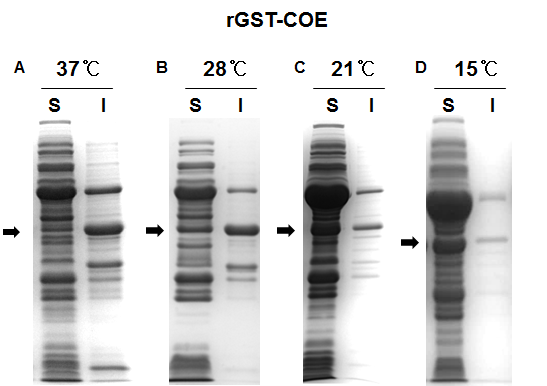


**Fig. S1. SDS-PAGE analysis of rGST-COE expression at various induction temperatures.** S, soluble fraction; I, insoluble fraction. The solid arrows indicate rGST-COE.


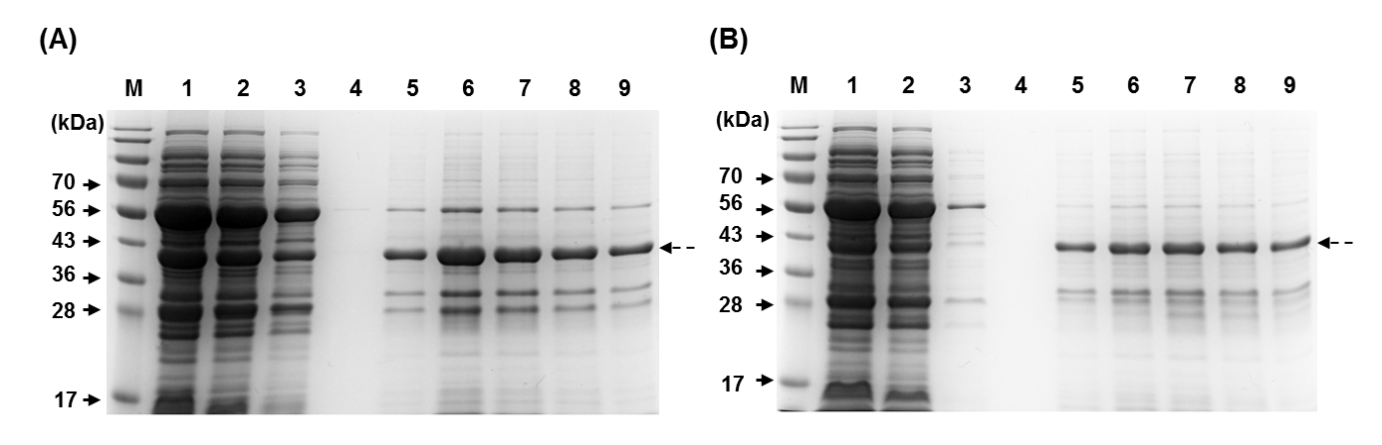


**Fig. S2.** **SDS-PAGE analysis of rGST-COE (A) and rGST-S1D (B) purified using glutathione sepharose 4B column.** Dotted arrow indicates rGST-COE and rGST-S1D. M, size markers in kDa; Lanes: 1, soluble crude proteins; 2, unbound fractions; 3-4, washing fractions; 5-9, elution fractions.

**
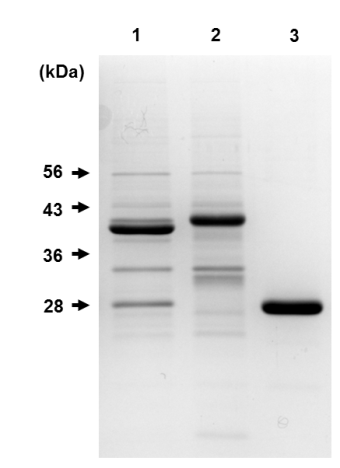
**

**Fig S3. SDS-PAGE analysis of purified rGST-COE, rGST-S1D and rGST. 1 µg of purified rGST-COE, rGST-S1D and rGST were loaded on the SDS-PAGE.** Lanes: 1, rGST-COE; 2, rGST-S1D; 3, rGST.
